# Carbolic Acid Injections in the Treatment of Carbuncle

**Published:** 1885-11

**Authors:** C. H. Wilkinson

**Affiliations:** Galveston, Texas


TREATMENT.

### CARBOLIC ACID INJECTIONS IN THE TREATMENT OF CARBUNCLE.

*By C. H. Wilkinson, M. D., Galveston, Texas.*

Read before the Galveston Medical Club. October 19th, 1885, and by that body voted to be published in Daniel's Texas Medical Journal.

THE subject which I have chosen to present for your consideration this evening, is one that I have given especial and unremitting attention to for the past six or eight years. In its presentation, therefore, I can confidently claim originality in its conception, and prolonged study in its evolution; furthermore, I will endeavor to be brief as possible in its rendition.

When we reflect upon the true nature of and important consequences involved in the pathological condition that I have alluded to in the caption of this article; the terrible sway of carbuncle when it has once obtained full mastery over the human system; the pain and anguish that invariably accompany its visitations, and its not unfrequent fatality; when we consider the long, unsatisfactory and uncertain, if not to say the frequently barbaric plan of treatment that often is adopted for the relief of this affliction, I believe that you will echo the sentiment that *any treatment whatsoever* that can substitute the plan heretofore pursued should be hailed by the profession as an advance in surgery, and be adopted as a boon to those unfortunate victims who occasionally suffer with this painful and destructive malady.

It has been my fortune in life—if to witness the miseries and misfortunes in others could be styled fortuitious—to encounter a large number of carbuncles in the persons of both hospital and private patients, and I can confidently assert that he who has not actually felt the hand of misery laid upon his own shoulder, or else witnessed this complaint in others, can form no idea of what suffering and anguish is comprehended under this generic term.

It is stated that one of the early Governors of Texas actually took his life with his own hands, when he learned that he was the victim of a second carbuncle, so great was his terror at the discovery, his first experience having proved so horrible; and who is it not familiar with the sad fate of some friend or prominent acquaintance by the hand of this same fell destroyer?

Carbuncle may be defined as a peculiar and unhealthy inflammation of the connective tissue of a part, characterized when fully developed, by great pain, redness and tumefaction of its periphery, perforation by small appertures of its center, extending as a rule in a circular direction, attended by rapid necrosis of cellular tissue, with septicæmia accompanying, and if unmolested by treatment ultimately terminating in somatic death.

It would appear that the inflammation of carbuncle was unlike all other varieties of inflammation known to surgery. Its *tumefied* periphery and multiple sinuosities through the skin contained in the area of its extension, certainly render it unlike any other form of disease that we are familiar with; while its proneness to unlimited extension, and its destructiveness to all the tissues that oppose its progress certainly characterize it as an inflammation of *unhealthy* nature. Hence, I repeat that carbuncle may be regarded as a *peculiar* and *unhealthy* inflammation of the connective tissue of a part; and I will add that in its proper treatment we must not expect to exercise control over its ravages by the use of plans employed in ordinary types of inflammation, but must look to agencies that while not severe and dangerous, exert a catalytic influence over the process that is going on.

The pain in this disease is always intense and unremitting; a slow, continued febrile state, differing with the various stages of the affection, always accompanies the morbid process, being sthenic at first but soon being characterized by adynamia as the disorder advances. It is truly a hectic, irritative or rather a septic fever in its more advanced stage, and it is very often marked by mild delirium from absorption of septic matter into the blood of the patient.

The tendency of carbuncle, if unmolested, is towards the death of its victim. In this respect it differs from most of its congeners in having no respect whatever for the "vis medicatrix naturæ." It continues to widen the circle of its destructiveness when once it has obtained a headway, and like a merciless fire it consumes everything in its way, leaving its smoke and ashes to escape from the system as best they can. Most of this debris finds exit through the numerous appertures in the superjacent skin, but the more subtle products of decomposition invariably gain access to the circulating fluid, causing thereby septicæmia and death of the patient, if not speedily arrested.

Hence the danger to life in this disease.

The cause of carbuncle is due, undoubtedly, to a sub-acute deterioration of the blood; usually to a retention therein of carbonacious material (such as occurs after typhoid fever and many other blood poisoning agencies), and *likewise* to an irritation applied to the skin, as through the chafing produced by clothing, etc. These two causes are generally required to produce the morbid condition under consideration, though often it appears without the recognition of the latter cause.

It occurs in every season of the year, attacks all classes of humanity over 30 years of age, and is not specially confined to either the indigent or the intemperate. It often happens twice in the same individual, and not unfrequently on different portions of the body at the same time.

Its duration may extend to several months, and its course, as said before, if unmolested, tends to death.

It is not so much a description of carbuncle, however, that I have invited your attention to this evening, but rather to a plan of treatment for the relief of this fearful malady, that, when instituted in time, is almost, if not absolutely certain to cure our patient, and that too, in a far quicker and less painful manner than by any other method that has heretofore been practiced. Formerly, either the knife, the cautery or an escharotic was the main reliance of surgeons in combatting this disease. That terrors were multiplied, and deaths actually produced by these methods of treatment, is beyond any question of doubt.

Nothing can be more painful, not to say more dangerous, than slashing through the sensitive and septic tissues of a carbuncle. Nothing can be less scientific in practice than the laying open thereby of large and swollen veins affording channels for the readier poisoning of the system by the introduction thereto of pyæmic and septicæmic foci.

The treatment which I propose to offer in lieu of that just criticised is simply the administrations, hypodermically, of carbolic acid, in the immediate vicinity of the most inflamed portions of the carbuncle. The process is simple, and may be relied upon in the great majority of instances wherein it is employed, as the following cases will testify.

In the summer of 1878, I was called to see a gentleman aged 52 years, who had been suffering with "carbuncle" for about three weeks. He was then delirious from the absorption of septic material, and in dispair of any relief had grown indifferent to his condition, and but for friends who urged the employment of a physician would soon have died from the virulence of his complaint.

I found upon his neck a carbuncle extending from ear to ear, and from the inferior occipital ridge to the seventh cervical vertebra. It measured fully six by seven inches in diameter and was suppurating slowly from at least a hundred little sinuses. All around the margin of the sore the skin was red and painful, and the slightest touch thereto would send a thrill of agony through his frame.

I injected into the most prominent sinuses a very strong solution of carbolic acid, and had the satisfaction of seeing a rapid restoration to health. So impressed was I with the value of carbolic acid in this individual instance, that I determined to make use of it in the treatment of all similar cases in the future.

I did not have to wait very long before an opportunity was presented me to try the remedy in its *full strength*. An Italian laborer aet 51 applied to me for treatment of a well developed carbuncle situated upon the posterior cervical region, which measured five by six inches and was very irritable. I selected two or three of the most prominent sinuses and injected into each of them about ten minims of pure carbolic acid, full strength. On the second day after the injection it was noticed that the inflamed areola had nearly disappeared, and the sore itself was casting off several large sloughs. A few more drops of acid were then injected, after which the ill-conditioned painful and highly inflamed carbuncle was converted into a simple, healthy, granulating ulcer, which healed to complete recovery within a few weeks after.

Since the treatment of this patient I have fallen heir to many similar cases; the carbuncle in the great majority of instances being situated on the nucha; and in every instance did a speedy return to health follow the carbolic acid treatment, as carried out in the case of the Italian. My method of employing the agent has been, as just stated, to select a few prominent sinuses and inject into them from five to ten drops of pure liquid carbolic acid, using an ordinary hypodermic, or, better still, a Heaton's hernia syringe, throwing the fluid in the direction of the hard, red and painful spots about the periphery, and being careful to pick up all excess of acid that might ooze back through neighboring sinuses, with sponge or blotting paper.

Carbolic acid in this class of cases acts by converting an unhealthy into a healthy inflammation. All erysipelatous tendency is checked instanter, wherever the acid touches, while the stimulus it affords to the capillaries promotes absorption on the one hand and healthy granulation on the other. Great sloughs of necrotic, connective tissue are thrown off, and the carbuncle is soon converted into a rapidly-healing simple ulcer.

Furthermore, carbolic acid acts as a local anæsthetic in these cases, and did it do no other good than this in these most painful affections we would be amply justified in its employment for this purpose alone.

I might consume your time a good while longer in the recital of cases wherein carbolic acid acted servicably in the cure of this complaint; but instances enough have been alluded to for my purpose, which is, gentlemen, merely to call your attention to this most valuable method in combatting one of nature's most horrible methods of annoying man.

It is safe in its execution, and, in comparison with other plans, is actually painless. It is quick and certain in its effects, and the acid being easily procurable, this treatment is always practicable.

There may be opponents to the plan I have proposed, and to the use of carbolic acid administered under the skin. But I submit that many of our most valuable discoveries in medicine have had the ordeal to run, and that it is no mark of inferiority in any remedy or plan of treatment in our profession to meet with opposition from some source or other during its probation.

In conclusion, gentlemen, I will venture the assertion that no man should ever die from carbuncle where the carbolic acid plan of treatment, as I have just advised its employment, has been carried out.

